# Dietary Adherence and Physical Activity in Adults with Type 2 Diabetes Mellitus in Southwest Saudi Arabia: A Cross-Sectional Study

**DOI:** 10.3390/nu18132170

**Published:** 2026-07-03

**Authors:** Nawaf W. Alruwaili, Hussain M. Alwadani, Nora Alafif, Aljazi Bin Zarah

**Affiliations:** 1 Department of Community Health Sciences, College of Applied Medical Sciences, King Saud University, Riyadh 11433, Saudi Arabia; 444105613@student.ksu.edu.sa (H.M.A.); nalafeef@ksu.edu.sa (N.A.); abinzaraah@ksu.edu.sa (A.B.Z.); 2 Najran Health Cluster, Najran 66255, Saudi Arabia

**Keywords:** type 2 diabetes mellitus, dietary adherence, physical activity, PDAQ, GPPAQ, Saudi Arabia, body mass index, glycaemic control

## Abstract

Background/Objectives: Dietary adherence and physical activity are pivotal yet understudied behavioral components of self-management of type 2 diabetes mellitus (T2DM) in the Middle East and North Africa region. This study aimed to quantify dietary adherence and physical activity levels, examine their association, and identify sociodemographic and clinical factors independently associated with these outcomes among adults with T2DM in southwest Saudi Arabia—a region chronically underrepresented in the literature. Methods: A descriptive cross-sectional study (n = 257; December 2023–March 2024) was conducted at a specialist diabetes center. The Perceived Dietary Adherence Questionnaire (PDAQ; 0–56 after removal of the fat-avoidance item with near-zero item-total correlation) and General Practice Physical Activity Questionnaire (GPPAQ) were administered alongside body mass index (BMI) and glycated hemoglobin (HbA1c) extracted from medical records. Bonferroni-corrected non-parametric bivariate tests, multiple linear regression with variance inflation factor diagnostics, and binary logistic regression were applied. Results: Mean 8-item PDAQ was 20.44 ± 10.04/56 (36.5%); carbohydrate spacing was the critical deficit (16.4%). GPPAQ distribution: 10.1% inactive, 28.0% moderately inactive, 49.0% moderately active, and 12.8% active, with sensitivity analysis ranging 28.0–47.5% in the two lowest categories. PDAQ–GPPAQ correlation was weak (Spearman r = 0.18; 95% CI: 0.06–0.29; r^2^ = 0.032). BMI alone accounted for 81.0% of PDAQ score variance (cross-sectional; direction of association not established; full model Adj. R^2^ = 0.826; LOO-CV R^2^ = 0.820, indicating model stability). Employment type showed the strongest cross-sectional association with GPPAQ-derived inactivity classification (housewife OR = 5.77; retired/seeking OR = 4.98 vs. employed), largely driven by the occupational component of the composite score. Conclusions: Dietary adherence was substantially below the maximum achievable score; BMI was the factor most strongly associated with PDAQ scores in cross-sectional analysis, though the direction of this relationship cannot be established. Physical activity levels were substantially associated with occupational patterns; housewives and retired/other participants faced approximately five-fold greater odds of being classified as inactive or moderately inactive compared with employed individuals. The weak PDAQ–GPPAQ correlation (r^2^ = 0.032) suggests these behaviors are not strongly co-determined and points to the potential value of distinct, hypothesis-generating intervention approaches for dietary quality and leisure-time physical activity in T2DM populations.

## 1. Introduction

Type 2 diabetes mellitus (T2DM) affects an estimated 537 million adults worldwide, with projections reaching 783 million by 2045 [1]. The Middle East and North Africa (MENA) region carries one of the highest age-adjusted prevalence rates globally; T2DM affects approximately 20–25% of Saudi adults, driven by rapid urbanization and adoption of sedentary, energy-dense dietary patterns [2]. Within the Kingdom of Saudi Arabia, a marked concentration of published research in the three metropolitan centers—Riyadh, Jeddah, and Madinah—leaves peripheral regions, including the southwest, chronically underrepresented in the epidemiological literature.

Dietary adherence and physical activity are the two most influential modifiable behavioral pillars of T2DM self-management. Suboptimal dietary adherence sustains hyperglycemia and accelerates vascular complications, while physical inactivity compounds insulin resistance and independently predicts premature mortality [3,4]. Despite international guidelines recommending at least 150 min of moderate-intensity aerobic activity per week and adherence to a nutrient-dense, low-glycemic diet [5,6], adherence rates in Saudi T2DM populations remain low [7,8]. These two behaviors are often examined in isolation; to our knowledge, their simultaneous assessment using multivariate analysis has not previously been reported in a southwest Saudi T2DM population, though unpublished or non-indexed studies may exist.

This study addresses that evidence gap by pursuing three objectives: (i) quantifying dietary adherence using the Perceived Dietary Adherence Questionnaire (PDAQ) and physical activity using the General Practice Physical Activity Questionnaire (GPPAQ); (ii) examining the correlation between these two behavioral outcomes; and (iii) identifying sociodemographic and clinical factors independently associated with these outcomes through multivariate regression. The findings are intended to inform the design of distinct, evidence-based primary care intervention streams for diet and physical activity in the region.

## 2. Materials and Methods

### 2.1. Study Design and Setting

A descriptive cross-sectional study was conducted in accordance with STROBE guidelines at a specialist Diabetes and Endocrinology Center within a tertiary public hospital in southwest Saudi Arabia (27 December 2023–10 March 2024).

### 2.2. Participants

A consecutive convenience sample of 257 adults was enrolled during scheduled outpatient visits. Inclusion criteria: confirmed T2DM diagnosis for ≥6 months, current antidiabetic pharmacotherapy, age ≥ 18 years, and permanent regional residence. Exclusion criteria: type 1 diabetes, insulin-only treatment (participants receiving insulin in combination with oral antidiabetic agents were eligible), T2DM diagnosis < 6 months, or inability to provide written informed consent.

### 2.3. Sample Size

All 257 adults who attended scheduled outpatient appointments during the 10-week recruitment window (27 December 2023–10 March 2024) and met the eligibility criteria were enrolled; no patient refused participation. The window was deliberately concluded before Ramadan (beginning late March 2024) to avoid seasonal confounding of dietary behavior. The sample size reflects the operational constraint of a single specialist center. The adequacy of this sample was evaluated through post hoc power analysis (G*Power 3.1). This post hoc analysis is provided as a descriptive sensitivity assessment only and does not replace an a priori sample size calculation, which was not performed given the operational census design (all eligible consecutive attendees enrolled). For the primary multiple linear regression model (Adj. R^2^ = 0.826, k = 7 predictors), the observed Cohen’s f^2^ = 4.73 yields power > 0.999 (α = 0.05), far exceeding the conventional 0.80 threshold. Power for the binary logistic regression was similarly robust: housewife versus employed (OR = 5.77) achieved power = 0.98, and retired/seeking versus employed (OR = 4.98) achieved power = 1.00. For the Spearman correlation (r = 0.18, α = 0.05), power was 0.80; the minimum detectable correlation at 80% power with n = 257 is r = 0.174, marginally above the observed value, which is appropriate given the descriptive and secondary role of this analysis. The single-center convenience design may overrepresent complex T2DM cases, which should be considered when generalizing results.

### 2.4. Data Collection Instruments

Perceived Dietary Adherence Questionnaire (PDAQ): A validated instrument originally comprising nine items scored 0–7 days per item. Item-level analysis revealed that Q9 (fat avoidance) had a near-zero corrected item-total correlation (r = −0.013, *p* = 0.833) and its removal increased Cronbach’s α from 0.611 to 0.643. Accordingly, the study-specific 8-item modified PDAQ (not fully equivalent to the original validated 9-item instrument; total range 0–56) was used for all analyses, assessing adherence to key dietary behaviors recommended for T2DM management [9]. The PDAQ was adapted into Arabic using forward–backward translation by two bilingual registered dietitians with T2DM clinical experience. The Arabic version was piloted in 15 T2DM outpatients not included in the main study; no items required modification. Content validity was then confirmed by five bilingual (Arabic–English) experts using a four-point relevance scale: I-CVI = 1.00 for all nine items and S-CVI/Ave = 1.00, exceeding accepted thresholds (≥0.80 and ≥0.90) [10]. Cronbach’s α = 0.643 for the 8-item scale; corrected item-total correlations ranged from 0.094 (Q4) to 0.448 (Q8, healthy oils). Removing Q9 increases α to 0.643, suggesting this item warrants review in future validations. The heterogeneous multi-domain structure of the PDAQ partly accounts for the relatively low α [9,10]. Items capture adherence to healthy eating plans, fruit and vegetable intake, glycemic-index carbohydrate choices, sugar avoidance, dietary fibre, carbohydrate spacing (distributing intake evenly across meals to attenuate postprandial glucose excursions), omega-3 intake, and oil quality. Fat avoidance (Q9) was excluded from the final scale as described above.

General Practice Physical Activity Questionnaire (GPPAQ): A validated six-item physical activity questionnaire [11]. Because the original GPPAQ does not provide a universal quantitative scoring algorithm, we applied a pragmatic MET-weighted composite score (Section 2.6) to derive a GPPAQ-derived classification. The resulting categories should be considered exploratory and are not directly comparable with standard GPPAQ outputs. A sensitivity analysis across three threshold sets was conducted to assess classification stability, though this does not constitute validation of the adapted scoring approach.

### 2.5. Clinical Variables

BMI was measured and categorized as normal/underweight (<25.0 kg/m^2^), overweight (25.0–29.9 kg/m^2^), or obese (≥30.0 kg/m^2^). HbA1c was extracted from the most recent medical record entry and dichotomized as good (<7%) or poor (≥7%) glycemic control per American Diabetes Association standards [12].

### 2.6. Statistical Analysis

Data was analyzed using IBM SPSS Statistics v25. Normality was assessed using the Kolmogorov–Smirnov test (KS = 0.082, *p* = 0.061) and the Shapiro–Wilk test (W = 0.967, *p* < 0.001; skewness = 0.60), confirming deviation from normality and justifying non-parametric approaches throughout. Group comparisons used the Mann–Whitney U test (two groups) and the Kruskal–Wallis H test (three or more groups). Bonferroni correction was applied across all 12 bivariate tests (corrected α = 0.0042). Spearman’s rank correlation with 95% confidence intervals (Fisher z-transformation) quantified the PDAQ–GPPAQ association.

The GPPAQ composite score was computed as follows: vigorous sport (×2.0) + cycling (×1.5) + pace-adjusted walking + housework (×0.75) + gardening (×1.0) + occupational MET-hours (0/1.0/3.5/7.0). Classification thresholds—Inactive = 0, Moderately Inactive > 0 to <2.5, Moderately Active 2.5 to <6.0, Active ≥ 6.0—were researcher-defined and evaluated through sensitivity analysis across conservative (1.5/4.0) and liberal (3.0/7.5) threshold sets. This sensitivity analysis demonstrates classification stability but does not constitute validation of the adapted scoring approach; this is acknowledged as a methodological limitation (Section 4.4).

Multiple linear regression examined PDAQ total score as the dependent variable with seven predictors: sex, urban residence, education (two dummies), BMI category (two dummies), and HbA1c. Variance inflation factors (VIF) and the design matrix condition number were computed to assess collinearity; a BMI-alone model and a BMI+HbA1c model were compared with the full model to quantify incremental explanatory value and to test the robustness of BMI coefficients under different covariate sets. To verify robustness against mild residual non-normality, bootstrap confidence intervals (2000 replications, bias-corrected) were computed for all regression coefficients. Leave-one-out cross-validation (LOO-CV) was used to assess predictive stability, as it provides more reliable estimates than k-fold CV when between-group separation is extreme (LOO-CV R^2^ = 0.820). Data was complete for all 257 participants; no missing values were present for any variable. Binary logistic regression examined GPPAQ-derived inactivity (Inactive or Moderately Inactive = 1) as a function of urban residence, education, age category, and employment type; Nagelkerke R^2^ was reported. A supplementary leisure-time-only logistic regression (excluding occupational MET-hours from the activity composite) was conducted to assess the potential circularity between employment type and the occupational component of the GPPAQ-derived classification.

### 2.7. Ethics

Ethical approval was granted by the Regional Health Ethics Committee (IRB No. 23122; approval date: 11 December 2023) and the King Saud University Institutional Review Board (IRB No. 238355; approval date: 11 December 2023). Written informed consent was obtained from all participants; procedures adhered to the Declaration of Helsinki.

## 3. Results

### 3.1. Participant Characteristics

A total of 257 adults with T2DM were enrolled (Table 1). The sample was predominantly male (65.4%), married (84.0%), aged >50 years (62.2%), and had ≤primary or intermediate schooling (51.4%). Urban residents comprised 52.5%. Clinically, 80.1% were overweight or obese, and 74.3% had poor glycemic control (HbA1c ≥ 7%).

### 3.2. Dietary Adherence (PDAQ)

Mean 8-item PDAQ total score was 20.44 ± 10.04/56, representing 36.5% of the maximum achievable score (Table 2). Adherence was highest for fruit and vegetable intake (52.1%) and omega-3-rich foods (44.7%) and critically lowest for carbohydrate spacing (16.4%) and avoidance of high-sugar foods (31.4%). Carbohydrate spacing (Q6) refers to distributing carbohydrate intake evenly across meals to attenuate postprandial glucose excursions [13]; it recorded the lowest adherence of all nine items (mean 1.15 ± 2.05 days/week; 16.4%), representing the most clinically actionable nutritional gap in this population (see also Supplementary File S2 for item-level distributions by BMI category).

### 3.3. Physical Activity (GPPAQ)

No participant reported sedentary desk work, consistent with the predominantly manual and non-employment occupational profile of this sample (Table 3). Leisure-time inactivity was high: 73.9% did no sport, 95.3% did no cycling, and 42.4% walked slowly. GPPAQ-derived classification under primary thresholds: 10.1% inactive, 28.0% moderately inactive, 49.0% moderately active, and 12.8% active. Overall, 61.9% were classified as at least moderately active; however, a substantial proportion of this activity reflects occupational MET-hours included in the composite score rather than leisure-time activity. Sensitivity analysis demonstrated consistent employment-type patterns across all threshold sets (proportion in the two lowest categories: 28.0–47.5%), indicating that the primary classification is not an artifact of threshold choice. To supplement the ordinal classification, the underlying MET-weighted continuous composite score was examined (mean 5.12 ± 4.34; range 0–28.5; coefficient of variation 84.9%), revealing substantial inter-individual variability in physical activity that the four-category ordinal scale partially obscures. The proportion classified as inactive or moderately inactive differed markedly by employment status: 4.8% of employed participants (n = 3/63), 44.0% of housewives (n = 22/50), and 35.4% of retired/other participants (n = 51/144). This difference is largely explained by the occupational component of the GPPAQ-derived classification and is interpreted descriptively.

### 3.4. PDAQ–GPPAQ Association

Spearman r = 0.18 (95% CI: 0.06–0.29, *p* = 0.004; r^2^ = 0.032). The weak correlation and low shared variance indicate that dietary adherence and physical activity are not strongly co-determined in this sample, though unmeasured shared influences cannot be excluded.

### 3.5. Bivariate Associations

Bivariate results with Bonferroni-corrected α = 0.0042 are presented in Table 4. PDAQ associations surviving correction: urban versus rural residence (17.29 vs. 23.92, *p* < 0.001), education level (primary 18.83 vs. diploma+ 24.23, *p* < 0.001), BMI (normal/UW 35.90 vs. obese 11.97, *p* < 0.001), and HbA1c (good 33.83 vs. poor 15.81, *p* < 0.001). The sex difference (male 19.24 vs. female 22.69, *p* = 0.016) did not survive Bonferroni correction. GPPAQ classification differed significantly by age (H = 24.64, *p* < 0.001), education (H = 17.09, *p* = 0.0002), and employment type (H = 23.63, *p* < 0.001). The GPPAQ median was 3 (IQR 2–3) for virtually every subgroup, indicating limited discriminative ability of the ordinal scale.

### 3.6. Multivariate Regression

Table 5 presents multivariate results. BMI category alone accounted for R^2^ = 0.810 of PDAQ score variance in this cross-sectional sample; a sensitivity analysis using continuous BMI midpoints yielded similarly high explanatory value, indicating that the high explained variance is not solely a categorical coding artifact. The full seven-predictor model reached Adj. R^2^ = 0.826 (F(7, 249) = 174.6, *p* < 0.001). The direction of the BMI–PDAQ association cannot be established in this cross-sectional design. Supplementary File S2 presents full PDAQ score distributions by BMI category at both the total and item levels. BMI–HbA1c collinearity (Spearman r = −0.744) produced elevated but sub-critical VIF (obesity VIF = 7.20, HbA1c VIF = 4.07, both below the conventional threshold of 10); the design matrix condition number was 14.65 (well below the problematic threshold of 30), suggesting that multicollinearity does not invalidate inference. A sensitivity analysis rerunning the model without HbA1c showed that BMI coefficients remained directionally consistent (overweight β = −8.96; obese β = −18.08). The HbA1c coefficient (β = +5.57) is best interpreted as a concurrent clinical marker associated with overall self-management, contributing only ΔAdj. R^2^ = 0.015 beyond BMI alone. BMI and HbA1c were measured concurrently with PDAQ; either may be a correlate, a consequence, or a broader self-management marker. The urban–rural coefficient (β = −1.04, *p* = 0.047) is exploratory and did not survive Bonferroni correction. Regression residuals showed mild non-normality (Shapiro–Wilk W = 0.972, *p* < 0.001; skewness = 0.36; kurtosis = 1.70); however, bootstrap 95% confidence intervals (2000 replications) were virtually identical to OLS intervals for all seven predictors, indicating inferential robustness. In the logistic model, employment type was most strongly associated with GPPAQ-derived inactivity classification (housewife OR = 5.77, 95% CI: 1.92–17.30, *p* = 0.002; retired/seeking OR = 4.98, 95% CI: 2.01–12.38, *p* < 0.001; Nagelkerke R^2^ = 0.192). A supplementary leisure-time-only analysis (excluding occupational MET-hours) found no significant association between employment type and leisure-time inactivity (housewife OR = 0.36, *p* = 0.458; retired OR = 1.09, *p* = 0.925; Nagelkerke R^2^ = 0.033), confirming that the main model finding is driven by the occupational component of the GPPAQ-derived classification and should be interpreted descriptively.

## 4. Discussion

To our knowledge, this study is among the first to assess dietary adherence and physical activity simultaneously with multivariate analysis in a T2DM cohort from southwest Saudi Arabia. Three principal cross-sectional findings emerge: dietary adherence was substantially below the maximum achievable score (36.5%), with BMI category the factor most strongly associated with PDAQ scores; GPPAQ-derived activity levels were substantially associated with occupational patterns, with employment type showing the strongest cross-sectional association with inactivity classification; and the low PDAQ–GPPAQ correlation (r^2^ = 0.032) suggests these behaviors are not strongly co-determined, which may inform hypothesis-generating intervention priorities.

### 4.1. Dietary Adherence

A mean 8-item PDAQ of 20.44/56 (36.5%; noting the borderline internal consistency of this study-specific modified scale, see Limitations) situates this cohort below the 38% reported in Nepal [14] and is consistent with Iranian data in which fewer than one-third of patients met dietary guidelines [15]. Alharbi et al. found that 62.2% of Saudi T2DM patients in Madinah had adequate dietary knowledge yet remained non-adherent [8], and Aljahdali and Bawazeer documented similar patterns in Riyadh outpatients [16], indicating that knowledge alone does not drive behavior change. Social pressure during communal mealtimes, affordability, and the complexity of carbohydrate management are documented barriers in regional settings that are likely to apply to this cohort [15,17,18].

The most critical specific deficit is carbohydrate spacing (16.4%), which generates large postprandial glucose excursions that independently drive HbA1c elevation and cardiovascular risk [13]. At the other end of the spectrum, omega-3 adherence (44.7%) [19] and fruit and vegetable intake (52.1%) [20] represent relative strengths. These strengths provide a counseling platform for targeted reinforcement rather than requiring wholesale dietary reform. BMI category was the factor most strongly associated with 8-item PDAQ scores in cross-sectional analysis (R^2^ = 0.810 for BMI alone; LOO-CV R^2^ = 0.820; full item-level distributions by BMI category in Supplementary File S2), with obese participants scoring 18.08 points lower than normal/underweight participants on average. The cross-sectional design precludes establishing the direction of this association: it is equally plausible that poor dietary adherence contributed to greater adiposity, that adiposity influenced dietary choices and self-reporting patterns, or that both reflect unmeasured self-management capacity. This bi-directionality is a key limitation of the current design [21,22].

Education was independently associated with dietary adherence (diploma+ 24.23 vs. ≤primary 18.83; *p* < 0.001), consistent with evidence that higher health literacy enables interpretation of food labels and understanding of the glycemic index [23]. Over half the sample had only primary schooling, underscoring the need for visual, non-literacy-dependent dietary communication strategies. The rural–urban gradient (rural 26.14 vs. urban 19.70) is consistent with evidence that ultra-processed urban food environments impair dietary quality [16,24], though this is an exploratory finding at *p* = 0.047 after Bonferroni adjustment.

### 4.2. Physical Activity

The corrected GPPAQ analysis reveals that 61.9% are at least moderately active, driven substantially by 22.6% in physically demanding occupations. However, occupational and leisure-time activities have distinct metabolic effects: structured leisure-time exercise stimulates GLUT-4 translocation and skeletal muscle insulin sensitivity most effectively [25,26], whereas Mutie et al. demonstrated that only leisure-time activity independently reduced T2DM risk [27]. The leisure-time data remain alarming: 73.9% performed no sport, 95.3% no cycling, and 42.4% walked slowly. Walking pace carries independent prognostic significance—Boonpor et al. reported 73–91% higher T2DM incidence in slow versus brisk walkers [28], and Hamasaki confirms that intensity, not merely duration, is the key counseling target [29].

Employment type showed the strongest cross-sectional association with GPPAQ-derived inactivity classification in the logistic model (housewife OR = 5.77; retired/seeking OR = 4.98); this association largely reflects the occupational component of the composite score and should be interpreted descriptively, consistent with findings by Alzahrani et al. in Saudi primary care settings [30] and by Taylor et al. across diverse low- and middle-income contexts [31]. These findings suggest that recreational activity prescriptions—including walking pace progression targets and structured home exercise—may be particularly relevant for this group [26,32]. Age > 60 showed a borderline association with inactivity (OR = 2.35, *p* = 0.076), consistent with physical decline trajectories in T2DM [33,34].

### 4.3. Independence of Behaviors and Intervention Implications

The weak PDAQ–GPPAQ correlation (r^2^ = 0.032) suggests that dietary adherence and physical activity are not strongly co-determined in this sample, which may support the hypothesis that parallel, separately tailored intervention approaches could be considered [35]. Holmen et al. reported stages of change at program entry [36], underscoring that specialist attendance does not automatically lead to self-management behavior change. Structured diabetes self-management education and support (DSMES) programs addressing exercise-specific barriers and dietary skills—particularly carbohydrate spacing—may be particularly valuable in this context [32]. Chan et al. demonstrated that family social support mediates self-efficacy and self-care in T2DM [37], a particularly salient mechanism given this cohort’s 84% married status.

### 4.4. Strengths and Limitations

Strengths include the following: simultaneous assessment with the 8-item PDAQ (Cronbach’s α = 0.643 after data-driven exclusion of a non-contributing item) and an adapted GPPAQ-derived classification; Arabic content validation by a five-expert panel (I-CVI = 1.00; S-CVI/Ave = 1.00) with forward–backward translation and a 15-patient pilot; multivariate regression with VIF, condition number, and leave-one-out cross-validation (LOO-CV R^2^ = 0.820) indicating model stability; bootstrap confidence interval validation (2000 replications) confirming inferential robustness; a leisure-time-only sensitivity analysis substantially clarifying (though not fully resolving) the employment–GPPAQ circularity concern, as the limitation of including occupational activity in the composite score is retained; three-threshold GPPAQ sensitivity analysis with continuous MET-weighted composite score; Bonferroni correction for multiple testing; and a focus on an underrepresented southwest Saudi T2DM population.

Limitations of this study include the following: (1) cross-sectional design, which precludes causal or directional inference and is particularly relevant for interpreting the BMI–PDAQ, HbA1c–PDAQ, and employment–inactivity associations; (2) single-center consecutive convenience sampling, which may overrepresent complex T2DM cases and limits generalizability to primary care or community-based populations; (3) researcher-adapted GPPAQ scoring with researcher-defined MET weights and thresholds—evaluated through three convergent analyses (three-threshold sensitivity analysis, continuous composite score, and leisure-time-only logistic regression) but not formally validated against an external criterion; (4) Arabic adaptation of the PDAQ with content validity established (I-CVI = 1.00; S-CVI/Ave = 1.00) but structural (factor-analytic) and criterion validity not yet published for Saudi T2DM populations; (5) borderline internal consistency of the PDAQ (Cronbach’s α = 0.611 before Q9 removal; α = 0.643 for the final 8-item scale), with structural and criterion validity not yet published for Saudi T2DM populations; (6) exclusive reliance on self-reported dietary and activity measures, mitigated in part by the use of objective BMI measurements and HbA1c values extracted from medical records as primary outcome correlates; (7) absence of accelerometry-based objective physical activity assessment; and (8) unmeasured confounders including diabetes duration, medication burden, comorbidities, depression, socioeconomic status, health literacy, and Ramadan-related behavioral variation.

## 5. Conclusions

In this cross-sectional sample of adults with T2DM in southwest Saudi Arabia, dietary adherence was substantially below the maximum achievable score (36.5%), and BMI category was the factor most strongly associated with PDAQ scores in cross-sectional analysis; the direction of this relationship cannot be established. GPPAQ-derived activity levels were substantially associated with occupational patterns; leisure-time inactivity was high, and non-employed individuals faced substantially greater odds of inactivity classification, largely reflecting occupational activity differences. The weak PDAQ–GPPAQ correlation suggests these behaviors are not strongly co-determined, which is a hypothesis-generating observation. These findings suggest potential priorities for (i) dietary counseling targeting carbohydrate spacing, particularly for individuals with obesity and poor glycemic control; and (ii) promotion of leisure-time physical activity among non-employed patients. These priorities require confirmation in longitudinal studies. Future research should employ longitudinal designs, accelerometry-based physical activity measurement, fully validated Arabic versions of the instruments, and community-based sampling to establish directional relationships and inform evidence-based intervention design in Saudi Arabia and the broader MENA region.

## Figures and Tables

**Table 1:** Participant characteristics and clinical profile (n = 257).

Variable/Category	n	%
Sex—Male/Female	168/89	65.4/34.6
Age—<30/30–49/50–60/>60 yrs	19/78/72/88	7.4/30.4/28.0/34.2
Marital status—Married/Other	216/41	84.0/16.0
Education—≤Primary/High school/Diploma+	132/44/81	51.4/17.1/31.5
Employment—Employed/Housewife/Retired–other	63/50/144	24.5/19.5/56.0
Residence—Urban/Rural	135/122	52.5/47.5
BMI—Normal-Underweight/Overweight/Obese	51/90/116	19.8/35.0/45.1
HbA1c—Good (<7%)/Poor (≥7%)	66/191	25.7/74.3

BMI, body mass index; HbA1c, glycated hemoglobin.

**Table 2:** PDAQ item-level scores (n = 257).

Q	Item	Mean ± SD	Adherence (%)
1	Following a healthy eating plan	2.14 ± 2.27	30.6
2	Eating recommended fruit and vegetable servings	3.65 ± 2.16	52.1
3	Eating low glycemic index carbohydrate foods	2.61 ± 2.20	37.3
4	Avoiding foods high in sugar	2.19 ± 2.35	31.4
5	Eating foods high in dietary fiber	3.12 ± 2.72	44.5
6	Spacing carbohydrates evenly throughout the day	1.15 ± 2.05	16.4
7	Eating fish or foods high in omega-3 fatty acids	3.13 ± 2.31	44.7
8	Using healthy oils (canola, olive, flax, walnut)	2.44 ± 2.64	34.9
9	Avoiding foods high in fat (excluded from final 8-item total)	2.32 ± 2.01	33.2
Total	Total 8-item PDAQ score (Q1–Q8)	20.44 ± 10.04	36.5

Each item scored 0–7 days/week. The final 8-item PDAQ total range is 0–56 after exclusion of Q9. Adherence (%) = (mean/7) × 100 for items; (20.44/56) × 100 for the 8-item total. Q9 is shown descriptively only and was not included in the final total score.

**Table 3:** GPPAQ physical activity profile and sensitivity analysis (n = 257).

Category	n	%
Occupational activity: sedentary desk work	0	0.0
Occupational activity: standing/walking most of the time	66	25.7
Occupational activity: definite physical effort	50	19.5
Occupational activity: vigorous physical activity	8	3.1
Not applicable (retired, housewife, unemployed)	133	51.8
GPPAQ overall classification (primary thresholds: 2.5/6.0)		
Inactive	26	10.1
Moderately inactive	72	28.0
Moderately active	126	49.0
Active	33	12.8
Sensitivity analysis: % in two lowest categories	n	%
Conservative thresholds (1.5/4.0)	72	28.0
Primary thresholds (2.5/6.0)—used throughout	98	38.1
Liberal thresholds (3.0/7.5)	122	47.5

GPPAQ scoring is a pragmatic MET-weighted adaptation (Section 2.6); thresholds are researcher-defined. PA, physical activity.

**Table 4:** Bivariate associations: PDAQ total score and GPPAQ classification (n = 257).

Variable	Category	n	PDAQ Mean ± SD [Median (IQR)]	*p* ^a^	GPPAQ Median (IQR)	*p* ^a^
Sex	Male/Female	168/89	19.24 ± 9.69/22.69 ± 10.37	0.016 ^b^	3 (2–3)/3 (2–3)	0.507
Age	<40/40–60/>60	42/127/88	21.12/21.06/19.22	0.326	3 (3–3)/3 (2–3)/2 (2–3)	<0.001 *
Residence	Urban/Rural	135/122	17.29 ± 8.84/23.92 ± 10.18	<0.001 *	3 (2–3)/3 (2–3)	0.132
Education	≤Primary/HS/Diploma+	132/44/81	18.83/18.25/24.23	<0.001 *	2 (2–3)/3 (3–3)/3 (2–3)	<0.001 *
Employment	Employed/HW/Retired	63/50/144	20.95/19.48/20.54	0.784	3 (3–3)/3 (2–3)/3 (2–3)	<0.001 *
BMI	Normal/UW/OW/Obese	51/90/116	35.90 ± 5.59/22.58 ± 3.59/11.97 ± 4.37	<0.001 *	3 (2–3)/3 (2–3)/3 (2–3)	0.237
HbA1c	Good/Poor	66/191	33.83 ± 6.31/15.81 ± 6.20	<0.001 *	3 (2–3)/3 (2–3)	0.090 ^c^

^a^ Mann–Whitney U (two groups) or Kruskal–Wallis H; Bonferroni-corrected α = 0.0042. * *p* < 0.0042 survives correction. ^b^ Effect sizes: rank-biserial r for MWU; η^2^ for KW. PDAQ statistics: Sex U = 5936, r = 0.21; Age H = 1.11, η^2^ = 0.000; Residence U = 5038, r = 0.39; Education H = 18.08, η^2^ = 0.063; Employment H = 0.04, η^2^ = 0.000; BMI H = 209.67, η^2^ = 0.818; HbA1c U = 12493, r = −0.98. ^b^ *p* = 0.016, does not survive Bonferroni; reported descriptively. ^c^ *p* = 0.090, does not survive correction. HW, housewife; OW, overweight; UW, underweight.

**Table 5:** Multivariate regression results: PDAQ total score (Panel A) and GPPAQ inactivity (Panel B) (n = 257).

Panel A—Linear Regression: PDAQ	β (95% CI)		Sig.	*p*		VIF	
Female sex (ref: male)	+0.82 (−0.26 to +1.91)		NS	0.137		1.16	
Urban residence (ref: rural) ^a^	−1.04 (−2.07 to −0.01)		†	0.047		1.15	
High school (ref: ≤primary)	+0.90 (−0.47 to +2.26)		NS	0.199		1.16	
Diploma+ (ref: ≤primary)	+0.77 (−0.39 to +1.93)		NS	0.192		1.26	
Overweight (ref: normal/UW)	−8.96 (−11.24 to −6.68)		***	<0.001		5.15	
Obese (ref: normal/UW)	−18.67 (−21.26 to −16.09)		***	<0.001		7.20 ^b^	
HbA1c good < 7% (ref: ≥7%) ^c^	+5.57 (+3.36 to +7.78)		***	<0.001		4.07 ^b^	
Model summary: BMI alone R^2^ = 0.810; BMI + HbA1c R^2^ = 0.825; full model Adj. R^2^ = 0.826; F(7, 249) = 174.6, *p* < 0.001.							
**Panel B—Logistic Regression: GPPAQ Inactivity ^D^**		**OR (95% CI)**			***p***		**Sig.**
Urban residence (ref: rural)		1.60 (0.91–2.83)			0.105		NS
High school (ref: ≤primary)		0.56 (0.23–1.36)			0.199		NS
Diploma+ (ref: ≤primary)		1.21 (0.57–2.59)			0.619		NS
Age 40–60 (ref: <40)		1.34 (0.54–3.29)			0.530		NS
Age > 60 (ref: <40)		2.35 (0.91–6.05)			0.076		†
Housewife (ref: employed)		5.77 (1.92–17.30)			0.002		**
Retired/seeking (ref: employed)		4.98 (2.01–12.38)			<0.001		***
Model summary: Nagelkerke R^2^ = 0.192; likelihood-ratio χ^2^ *p* < 0.001.							

^a^ Exploratory; does not survive Bonferroni correction. ^b^ Elevated VIF: BMI–HbA1c Spearman r = −0.744. ^c^ Collinear; R^2^ increment = 0.015 beyond BMI alone. ^D^ Inactive or Moderately Inactive (=1) vs. more active (=0). NS, not significant; † *p* < 0.10; ** *p* < 0.01; *** *p* < 0.001.

## Data Availability

The anonymized dataset supporting the findings of this study is available from the corresponding author (N.W.A.) upon reasonable request.

## Supplementary Materials

The following supporting information can be downloaded at: https://www.mdpi.com/article/10.3390/nu18132170/s1, Supplementary File S1: STROBE Checklist for Cross-Sectional Studies; Supplementary File S2 for PDAQ Scores by BMI Category.

## Abbreviations

ADA: American Diabetes Association; BMI: body mass index; CI: confidence interval; DSMES: diabetes self-management education and support; GLUT-4: glucose transporter type 4; GPPAQ: General Practice Physical Activity Questionnaire; HbA1c: glycated hemoglobin; IQR: interquartile range; IRB: Institutional Review Board; MET: metabolic equivalent of task; MENA: Middle East and North Africa; OR: odds ratio; PDAQ: Perceived Dietary Adherence Questionnaire; SD: standard deviation; STROBE: Strengthening the Reporting of Observational Studies in Epidemiology; T2DM: type 2 diabetes mellitus; VIF: variance inflation factor.
