# Supplementary material for: Dietary Adherence and Physical Activity in Adults with Type 2 Diabetes Mellitus in Southwest Saudi Arabia: A Cross-Sectional Study
Source: Nutrients. 2026 Jul 3;18(13):2170. doi: 10.3390/nu18132170 (PMC13364105; doi:10.3390/nu18132170)
Supplement: Supplementary file 1 [file nutrients-18-02170-s001.zip › nutrients-4396636-File S1.pdf]

Supplementary File S1: STROBE Checklist for Cross-Sectional Studies

Manuscript: Dietary Adherence and Physical Activity in Adults with T2DM in Southwest Saudi Arabia: A Cross-Sectional Study

Authors: Alruwaili N.W., Alwadani H.M., Alafif N., Bin Zarah A. | Journal: Nutrients (MDPI) | References: 38 | Tables: 5

Item	No.	Recommendation	Reported on page/location
**Title & abstract**			
Title and abstract	1	(a) Study design in title or abstract; (b) informative and balanced abstract	(a) Study design stated in abstract Methods: "descriptive cross-sectional study"; also reflected in revised title with "A Cross-Sectional Study" suffix. (b) Structured abstract: Background/Objectives, Methods, Results, Conclusions. Includes 95% CI for Spearman r; R^2 model comparison; GPPAQ sensitivity range; Bonferroni correction noted. Word count: ~250 words.
**Introduction**			
Background/rationale	2	Scientific background and rationale for investigation	Introduction §1–2: global T2DM burden [1], Saudi prevalence [2], lifestyle self-management rationale [3–6], Saudi adherence gaps [7,8]. Southwest Saudi Arabia's underrepresentation is explicitly stated.
Objectives	3	State-specific objectives	Introduction §3: objectives (i) quantify PDAQ/GPPAQ, (ii) examine PDAQ–GPPAQ association, (iii) identify independent determinants through multivariate regression.
**Methods**			
Study design	4	Present key elements of the study design early in the paper	Section 2.1: descriptive cross-sectional; STROBE guidelines followed. Stated in abstract, Methods, and manuscript title.
Setting	5	Setting, locations, and relevant dates, including dates of recruitment	Section 2.1: specialist Diabetes and Endocrinology Center, tertiary public hospital, southwest Saudi Arabia; 27 December 2023–10 March 2024.
Participants	6	Eligibility criteria, sources, and methods of selection of participants	Section 2.2: inclusion — confirmed T2DM ≥6 months, antidiabetic treatment, age ≥18 years, permanent regional residence. Exclusion — type 1 diabetes, insulin-only, diagnosis <6 months, inability to consent. Consecutive convenience sample.
Variables	7	Clearly define all outcomes, exposures, predictors, potential confounders, and effect modifiers; give diagnostic criteria, if applicable	Sections 2.4–2.5: PDAQ (9-item, 0–63) with Arabic content validation by a five-expert panel (I-CVI = 1.00; S-CVI/Ave = 1.00) [9,10]; GPPAQ (6-item, 4 categories) [11]; BMI (3 categories per WHO); HbA1c (dichotomized per ADA 2024 [12]).
Data sources/measurement	8	For each variable of interest, give sources of data and details of assessment methods; describe comparability of assessment methods if there is more than one group	Sections 2.4–2.5: PDAQ and GPPAQ administered face-to-face by trained research assistants using a pre-tested Arabic questionnaire. BMI: measured height and weight. HbA1c: most recent value from medical records.

Bias	9	Describe any efforts to address potential sources of bias	Trained research assistants; standardized face-to-face interview; pre-tested questionnaire; two internationally validated instruments; Bonferroni correction for multiple testing; VIF for collinearity. Convenience sampling bias is acknowledged in Section 4.4.
Study size	10	Explain how the study size was arrived at	Section 2.3: n = 257 = all eligible consecutive attendees during the 10-week data collection window (operational constraint of a single specialist center). Sample adequacy established through post-hoc power analysis (G*Power 3.1): (i) primary multiple linear regression (R^2 = 0.856, k = 7): Cohen's f^2 = 5.94, power > 0.999; (ii) logistic regression — housewife OR = 5.77: power = 0.98; retired/seeking OR = 4.98: power = 1.00; (iii) Spearman correlation (r = 0.16, α = 0.05): power = 0.73; minimum detectable r at 80% power = 0.174 (secondary/descriptive analysis). No a priori formula used; sample size reflects full-census consecutive enrollment.
Quantitative variables	11	Explain how quantitative variables were handled in the analyses; if applicable, describe which groupings were chosen and why	Section 2.5: BMI 3 categories (<25.0, 25.0–29.9, ≥30.0 kg/m²); HbA1c dichotomized (<7%/≥7%). Section 2.6: GPPAQ composite MET-weighted score; classification thresholds defined and validated by sensitivity analysis across three threshold sets.
Statistical methods	12	(a) All statistical methods, including those used to control for confounding; (b) methods used to examine subgroups and interactions; (c) how missing data were addressed; (d) if applicable, methods for sensitivity analyses	(a) Normality: KS=0.082 (p=0.061), SW W=0.967 (p<0.001, skewness=0.60). Non-parametric throughout: Mann–Whitney U, Kruskal–Wallis H. Bonferroni-corrected α=0.0042 (12 tests). Spearman r with 95% CI. OLS with VIF and design matrix condition number (14.65, confirming sub-critical collinearity). Bootstrap CIs (2,000 replications, bias-corrected) computed for all regression coefficients; OLS and bootstrap CIs are virtually identical, confirming robustness. Sensitivity analysis: model rerun without HbA1c confirms BMI coefficients directionally identical. Binary logistic with Nagelkerke R^2. (b) Subgroup analyses by sex, age, residence, education, employment, BMI, HbA1c for both outcomes (Table 4). (c) No missing data. (d) GPPAQ sensitivity analysis: three threshold sets (conservative 1.5/4.0; primary 2.5/6.0; liberal 3.0/7.5); continuous MET-weighted composite score (mean 5.12 ± 4.34; CV = 84.9%) supplements ordinal classification.
**Results**			
Participants	13	Report numbers in each stage of study; give reasons for non-participation at each stage; consider use of a flow diagram	Section 3.1: 257 consecutive eligible attendees enrolled. All eligible patients were enrolled during the window; no staged exclusions were applicable to the consecutive design. Absence of a flow diagram is acknowledged as a limitation.
Descriptive data	14	(a) Give characteristics of study participants; (b) indicate the number of participants with missing data for each variable of interest	(a) Table 1: sex, age, marital status, education, employment, residence, BMI, HbA1c. (b) No missing data for any variable.
Outcome data	15	Report the number of outcome events or summary measures	Table 2: PDAQ item-level scores (mean ± SD; adherence %). Table 3: GPPAQ occupational distribution and classification [11]; sensitivity analysis across 3 threshold sets; continuous MET-weighted composite score distribution (mean 5.12 ± 4.34).

Main results	16	(a) Give unadjusted estimates and, if applicable, confounder-adjusted estimates; (b) report category boundaries; (c) if relevant, consider translating estimates of relative risk into absolute risk for a meaningful time period	(a) Table 4: bivariate (Mann–Whitney U, Kruskal–Wallis) with Bonferroni-corrected p-values. Table 5 Panel A: OLS β (95% CI), p, VIF; R^2 comparisons. Table 5 Panel B: logistic OR (95% CI), p, Nagelkerke R^2. (b) BMI: <25.0, 25.0–29.9, ≥30.0 kg/m²; HbA1c: <7%/≥7%; GPPAQ thresholds in Section 2.6. (c) Not applicable.
Other analyses	17	Report other analyses done — e.g., analyses of subgroups and interactions, and sensitivity analyses	Table 4: subgroup analyses by sex, age, residence, education, employment, BMI, HbA1c for both outcomes. Table 3 (sensitivity): GPPAQ across conservative/primary/liberal thresholds (28.0–47.5%). Section 3.6: BMI-alone R^2=0.840 vs full Adj. R^2=0.856; condition number=14.65; bootstrap 95% CIs confirm OLS robustness; sensitivity model without HbA1c confirms BMI dominance.
**Discussion**			
Key results	18	Summarise key results with reference to study objectives	Section 4 §1: all three objectives addressed—dietary adherence 36.1%; GPPAQ occupationally driven, employment type dominant predictor; r^2=0.026 confirming behavioral independence.
Limitations	19	Discuss limitations of the study, taking into account sources of potential bias or imprecision; discuss both direction and magnitude of any potential bias	Section 4.4: cross-sectional design precluding causal inference; single-center convenience sampling potentially overrepresenting complex T2DM cases and limiting generalizability to community-based populations; absence of a participant flow diagram; unmeasured confounders (diabetes duration, medication burden, depression, health literacy). NOTE: Previously listed limitations (PDAQ α, GPPAQ ceiling, BMI–HbA1c collinearity, residual non-normality) have been methodologically resolved in the revised manuscript and removed from the limitations section.
Interpretation	20	Give a cautious overall interpretation of results, considering objectives, limitations, multiplicity of analyses, and other studies	Section 4: 4 subsections with cautious interpretation, explicit limitation acknowledgments, and comparison with global/regional literature. Causal inference explicitly disclaimed. Exploratory findings flagged (urban–rural gradient, p=0.047). Conclusions (Section 5): specific and actionable.
Generalisability	21	Discuss the generalisability (external validity) of the study results	Section 4.4: single-center tertiary convenience sample may overrepresent complex T2DM; results not directly extrapolatable to primary care or community-based populations. Contributes region-specific evidence for southwest Saudi Arabia absent from the MENA literature.
**Other information**			
Funding	22	Give the source of funding and the role of the funders for the present study	Funding section: Ongoing Research Funding Program, King Saud University (ORF-2026-1562). Funder had no role in study design, data collection, analysis, interpretation, or writing.

Reference: von Elm E, Altman DG, Egger M, et al. The Strengthening the Reporting of Observational Studies in Epidemiology (STROBE) statement: guidelines for reporting observational studies. Lancet. 2007;370:1453–1457.
