# Supplementary material for: Dietary Adherence and Physical Activity in Adults with Type 2 Diabetes Mellitus in Southwest Saudi Arabia: A Cross-Sectional Study
Source: Nutrients. 2026 Jul 3;18(13):2170. doi: 10.3390/nu18132170 (PMC13364105; doi:10.3390/nu18132170)
Supplement: Supplementary file 1 [file nutrients-18-02170-s001.zip › nutrients-4396636-File S2.pdf]

Supplementary File S2
8-Item PDAQ Scores by BMI Category (n = 257)

Dietary adherence and physical activity in adults with type 2 diabetes mellitus in southwest Saudi Arabia: a cross-sectional study

PDAQ Item / Domain	Normal/Underweight (n=51)	Overweight (n=90)	Obese (n=116)
	Mean ± SD (Adherence %)	Mean ± SD (Adherence %)	Mean ± SD (Adherence %)
**A. PDAQ Total Score (range 0–56; Q1–Q8 summed)**			
Total PDAQ score	35.90 ± 5.59 (64.1%)	22.58 ± 3.59 (40.3%)	11.97 ± 4.37 (21.4%)
Median (IQR)	36.0 (31.5–39.5)	22.5 (20.0–25.0)	13.0 (9.0–15.0)
Range (min–max)	27–52	14–29	1–20
Kruskal–Wallis (overall)	H = 209.67, df = 2, $p < 0.001$, $\eta^2 = 0.818$		
**B. Item-Level Mean ± SD (Adherence % = Mean/7 × 100)**			
Q1 Following a healthy eating plan	4.33 ± 2.30 (61.9%)	2.20 ± 2.05 (31.4%)	1.14 ± 1.68 (16.3%)
Q2 Fruit and vegetable intake	4.82 ± 2.13 (68.9%)	4.11 ± 2.10 (58.7%)	2.78 ± 1.86 (39.7%)
Q3 Low glycaemic index carbohydrates	4.63 ± 2.37 (66.1%)	2.66 ± 1.99 (38.0%)	1.69 ± 1.62 (24.1%)
Q4 Avoiding high-sugar foods	3.63 ± 2.72 (51.9%)	2.13 ± 2.09 (30.4%)	1.61 ± 2.10 (23.0%)
Q5 High dietary fibre foods	5.69 ± 1.97 (81.3%)	3.72 ± 2.51 (53.1%)	1.52 ± 2.02 (21.7%)
Q6 Carbohydrate spacing [a]	3.27 ± 2.65 (46.7%)	1.12 ± 1.87 (16.0%)	0.23 ± 0.88 (3.3%)
Q7 Omega-3 fatty acids [19]	4.76 ± 2.11 (68.0%)	3.56 ± 2.23 (50.9%)	2.08 ± 1.91 (29.7%)
Q8 Healthy oils (canola, olive, flax)	4.76 ± 2.67 (68.0%)	3.08 ± 2.48 (44.0%)	0.93 ± 1.62 (13.3%)
**C. Regression Context (from Table 5, Panel A)**			
Overweight vs Normal/UW (β)	Ref	−8.96 (95% CI: −11.41, −6.51)	—
Obese vs Normal/UW (β)	Ref	—	−18.08 (95% CI: −20.86, −15.31)
Full model Adj. $R^2 = 0.826$; BMI-alone $R^2 = 0.810$; LOO-CV $R^2 = 0.820$			

Notes. PDAQ, Perceived Dietary Adherence Questionnaire. BMI, body mass index (WHO categories: Normal/Underweight < 25.0 kg/m²; Overweight 25.0–29.9 kg/m²; Obese ≥ 30.0 kg/m²). Each item scored 0–7 days/week; Q9 (fat avoidance) excluded after near-zero corrected item-total correlation ($r = -0.013$, $p = 0.833$). 8-item total range: 0–56. Adherence (%) computed as (mean/7) × 100 for individual items and (total/56) × 100 for total score. Group differences tested by Kruskal–Wallis H test; effect size reported as $\eta^2 = (H - k + 1)/(n - k)$. LOO-CV, leave-one-out cross-validation. Means and standard deviations are presented alongside medians and IQRs for total scores because non-parametric tests were used for inference.

[a] *Carbohydrate spacing = distributing carbohydrate intake evenly across meals to attenuate postprandial glucose excursions. This item recorded the lowest adherence of all eight items across all three BMI categories.*

[19] Natto, Z.S. et al. Omega-3 fatty acids effects on inflammatory biomarkers and lipid profiles among diabetic and cardiovascular disease patients. Sci. Rep. 2019, 9, 18867.
